# Functional interrogation of neuronal connections by chemoptogenetic presynaptic ablation

**DOI:** 10.1126/sciadv.aeb6755

**Published:** 2026-04-29

**Authors:** Hariom Sharma, Jennifer M. Panlilio, Madalina A. Robea, Noel H. McGrory, Daniel C. Bazan, Edward A. Burton, Harold A. Burgess

**Affiliations:** ^1^ Division of Developmental Biology, *Eunice Kennedy Shriver* National Institute of Child Health and Human Development, Bethesda, MD, USA.; ^2^ Pittsburgh Institute for Neurodegenerative Diseases, University of Pittsburgh, Pittsburgh, PA, USA.; ^3^ Department of Neurology, University of Pittsburgh, Pittsburgh, PA, USA.; ^4^ Pittsburgh VA Healthcare System, Geriatric Research, Education, and Clinical Center, Pittsburgh, PA, USA.

## Abstract

Most neurons are embedded in multiple circuits, with signaling to distinct postsynaptic partners playing functionally different roles. The function of specific connections can be interrogated using synaptically localized optogenetic effectors; however, these tools are often experimentally difficult to validate or produce paradoxical outcomes. We have developed a system for photoablation of synaptic connections originating from genetically defined neurons based on presynaptic localization of the fluorogen-activating protein dL5** that acts as a photosensitizer when bound to a cell-permeable fluorogen dye. Using the well-mapped zebrafish escape circuit as a readout, we first show that cytoplasmically expressed dL5** enables efficient spatially targeted neuronal ablation using near-infrared light. We then demonstrate that spatially patterned illumination of presynaptically localized dL5** (syp-dL5) can effectively disconnect neurons from selected downstream partners, suppressing postsynaptic responses and producing precise behavioral deficits. This technique should be applicable to almost any genetically tractable neuronal circuit, enabling refined manipulation of functional connectivity within the nervous system.

## Teaser

Patterned illumination of a presynaptically localized photosensitizer disconnects neurons from selected downstream partners.

## INTRODUCTION

Genetically encoded proteins that provide experimental control over neuronal activity have revolutionized neuroscience, revealing how specific neuronal cell types contribute to circuit function and behavior. These tools use gene regulatory elements to drive selective expression in cell types of interest, but the complexity of neuronal ramifications can make it difficult to resolve the function of downstream connections. For example, in the *Drosophila* brain, neurons have a median of 13 postsynaptic partners, but the CT1 visual interneuron connects to 6329 downstream targets (*1*). Thus, even manipulations that achieve exquisite target cell specificity are likely to influence multiple downstream postsynaptic partners.

To restrict experimental manipulations to desired output pathways, effector proteins can be fused to synaptic localization signals, allowing specificity through spatially controlled delivery of an activating chemical or light (*2*–*4*). For example, stimulation of channelrhodopsin in axons can restrict activation to a specific output pathway (*5*, *6*), while presynaptic expression of optogenetic inhibitors can selectively suppress transmission to downstream neurons (*7*, *8*). These methods are ideally suited to the optically transparent larval zebrafish system but would require animals to be immobilized under a microscope to allow for spatially patterned illumination precluding their use in freely swimming assays.

Fusion of a genetically encoded photosensitizer, miniSOG, to vesicle release proteins was previously used to achieve long-lasting synaptic inactivation in *Caenorhabditis elegans* (*9*). MiniSOG generates destructive singlet oxygen by binding flavin mononucleotide, which is ubiquitously present in cells. Exposure to 480-nm light led to a rapid reduction in synaptic currents that persisted for several hours after discontinuation of illumination. In this system, suppression is likely due to local destruction of vesicle proteins, with recovery occurring as new components are trafficked to the synapse. We were inspired by this system to develop an analogous method for synaptic ablation in zebrafish, using a recently developed photosensitizer that is substantially more effective than miniSOG, to enable behavioral experiments in larvae after the removal of selected synaptic connections.

The fluorogen-activating protein dL5** (herein dL5) is a synthetic fusion of two single-chain antibody subunits that binds with high affinity to an iodine-substituted malachite green fluorogen (MG-2I). The complex generates singlet oxygen at high quantum yield when illuminated (*10*–*12*). The dL5 system is more efficient than other phototoxic alternatives (*13*) and has already been used successfully to target mitochondria in zebrafish neurons, resulting in their bioenergetic collapse, depolarization, and cell death (*14*). Neither dL5 protein nor free MG-2I dye is photosensitizing alone, facilitating rearing under normal illumination conditions and allowing analysis using tests that require visual stimulation. The complex is activated by near-infrared light (NIR; 666 nm), which has excellent tissue penetration. Moreover, dL5 efficiently lesions cells when targeted to a variety of subcellular locations, including the nucleus, cytoplasm, mitochondria, or cell membrane (*12*). This raised the possibility of fusing dL5 to a vesicle protein and destroying specific synaptic outputs from a neuron via selective illumination, thus enabling studies that require long-term inactivation of connections.

In this study, we confirmed that cytoplasmic dL5 can be used to lesion neurons using spatially patterned illumination. Then, by fusing dL5 with synaptophysin, we directed the trafficking of dL5 to synapses and enabled the selective ablation of output synapses from neurons of interest. We validated the syp-dL5 method through behavioral assessment of auditory escape and prepulse inhibition (PPI), taking advantage of the detailed mechanistic understanding of these circuits. This tool will be broadly useful in genetically tractable experimental systems, providing a means to interrogate the functional contributions of specific synapses through their persistent elimination.

## RESULTS

### Wide-field light exposure efficiently ablates dL5-expressing neurons

**Fig. 1.**
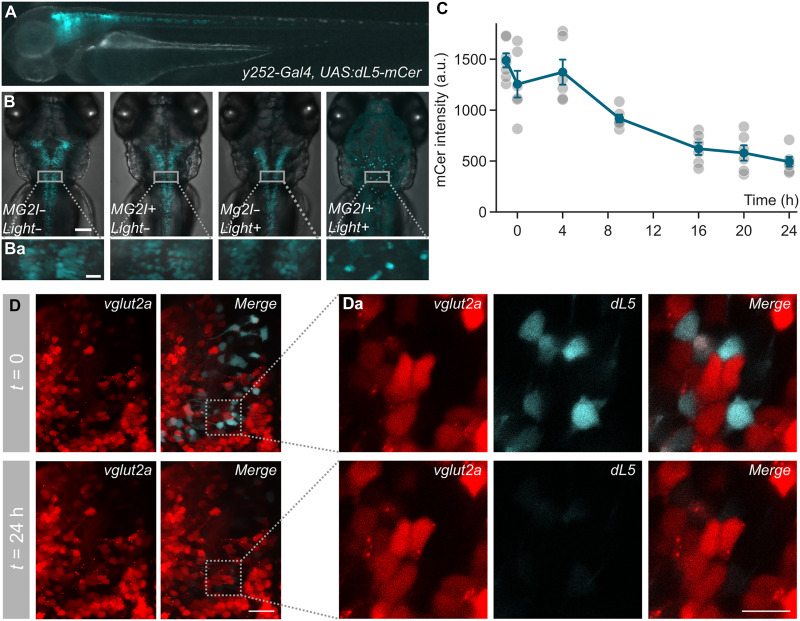
Efficient neuronal ablation using cytoplasmic dL5. (**A**) Transgenic *y252-Gal4, UAS:dL5-mCer* larva at 3 dpf. Merged fluorescent and visible light exposures. (**B**) Maximum projection of confocal images of *y252-Gal4, UAS:dL5-mCer* 6 dpf larvae. Untreated (left), MG-2I only, light only, and combined light and MG-2I as indicated. Puncta in ablated larvae are cellular debris. Scale bar, 100 μm. (Ba) A square ROI was selected from each panel in (B) and is presented as higher-magnification images in (Ba). Scale bar, 20 μm. (**C**) Cerulean fluorescence intensity (a.u., arbitrary units) in *y252-Gal4, UAS:dL5-mCer* immediately before and after photoablation and at the indicated time points. Points show values from individual larvae normalized to nonfluorescent sibling larvae. h, hours. (**D**) Maximum projection (D) and matched single horizontal confocal slices (Da) through part of the diencephalon of *y252:Gal4, UAS:dL5-mCer, vglut2a:DsRed* larvae immediately before exposure to NIR light (top panels) and 24 hours later (bottom panels). The region in (Da) is rotated from the boxed area in (D). Scale bars, 20 μm.

Previous studies have shown that cytoplasmic dL5 efficiently ablates zebrafish cardiac cells and that mitochondrially targeted dL5 enables neuronal ablation (*12*, *14*). To enable flexible cell type–specific expression, we generated a transgenic line with cytoplasmic dL5 expression under the control of an upstream activation sequence (UAS) promoter (Fig. 1A). We tested the ablation of cells expressing dL5 using the Gal4 line *y252-Gal4*, because ablation of neurons labeled in this line has multiple well-characterized effects on behavior (*15*–*17*). In *y252-Gal4, UAS:dL5-mCer* larvae that were treated with MG-2I for 24 hours and then exposed to 550 mW/cm^2^ wide-field (i.e., whole-body) 656-nm light for 20 min, we saw a complete loss of mCer-expressing cells at 24 hours (Fig. 1B and fig. S1A). Both MG-2I treatment and intense illumination were required for ablation (Fig. 1B). At 4 hours after light exposure, mCer-expressing cells remained intact—demonstrating that the loss of fluorescence is not due to photobleaching—but by 16 hours, near-total loss was observed (Fig. 1C). For wild-type embryos not expressing dL5, MG-2I treatment was nontoxic, including in embryos exposed to intense light, as evidenced by the presence of an inflated swim bladder at 6 days post-fertilization (dpf), which is a sensitive marker of health in zebrafish larvae (fig. S1B). To further assess potential effects of prolonged MG-2I exposure on neural circuit function, we examined spontaneous locomotion in dL5-expressing larvae treated with MG-2I in the absence of NIR illumination. No differences in swimming behavior were observed compared to untreated controls, either after drug washout (fig. S2A) or during continued exposure (fig. S2B). We tested different durations of light exposure and found a dose-response effect, with 20 min required for the complete loss of *y252-Gal4* neurons (fig. S1C).

Singlet oxygen has limited diffusion in a cellular environment, which should help to limit collateral damage during photoablation. In zebrafish, photostimulation of MG-2I treated mitochondrially targeted dL5-damaged mitochondria without acutely affecting the adjacent endoplasmic reticulum or Golgi apparatus (*14*). However prolonged illumination of membrane-localized dL5-expressing cells can damage neighboring cells (*12*). We therefore assessed the specificity of dL5 neuronal ablation by imaging cells adjacent to dL5-expressing cells in the same larva before exposure to MG-2I and NIR and 24 hours later. We focused on a diencephalic region in *y252-Gal4, UAS:dL5-mCer, vglut2a:DsRed* larvae, where *y252*-expressing neurons and *vglut2a*-expressing neurons are relatively sparse and rarely overlap. There was no loss of *vglut2a:DsRed* signal in this region after photoablation (Fig. 1D), and inspection of single confocal planes demonstrated that *vglut2a:DsRed* neurons that were closely apposed to *y252-Gal4, UAS:dL5-mCer* neurons remained intact despite the complete loss of dL5 neurons (Fig. 1Da).

**Fig. 2.**
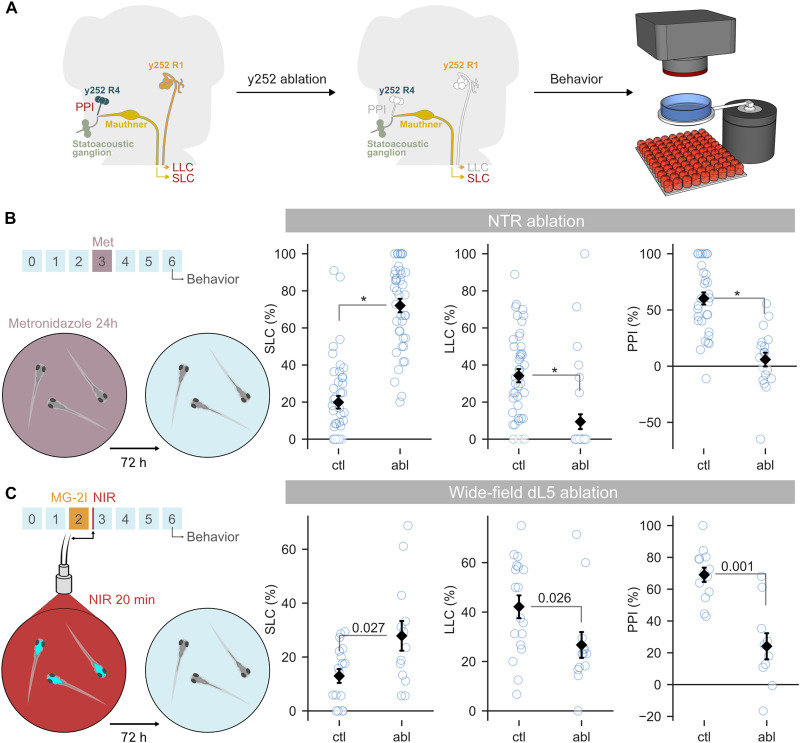
dL5 photoablation reproduces behavioral deficits caused by NTR ablation. (**A**) Relevant neurons for SLC and LLC escape behavior and PPI. *y252-Gal4* labels R1 neurons required for LLC responses and R4 neurons that mediate PPI. After ablation at 3 dpf, larvae were tested at 6 dpf for escape behavior and PPI using auditory/vibrational stimuli. (**B**) Increased SLC responses [*n* = 43 and 41 for ctl (NTR^−^/MTZ^+^) and abl (NTR^+^/MTZ^+^)], decreased LLC responses (*n* = 43 and 34), and reduction in PPI (*n* = 32 and 19) after NTR-mediated ablation of *y252* neurons. MWU, *P < 0.001. (**C**) SLC and LLC escape responses [both *n* = 18 and 13 for ctl (dL5^−^/Mg2I^+^) and abl (dL5^+^/Mg2I^+^)] and PPI (*n* = 13 and 10) in control and photoablated dL5-expressing *y252* neurons. MWU, *P* values indicated.

Next, we checked whether photoablation with dL5 reproduces behavioral changes resulting from ablation using the well-established nitroreductase (NTR) ablation method (*18*). NTR is a bacterial protein that converts the prodrug metronidazole into a cell-impermeant toxin, enabling cell-specific ablation (*19*). For these experiments, we used the *y252-Gal4* line, which labels neurons that contribute to escape behavior and sensorimotor gating (Fig. 2A). We ablated NTR-expressing *y252-Gal4* neurons with metronidazole treatment from 3 to 4 dpf and then tested larvae at 6 dpf. As previously reported, we observed an increase in short-latency C-start (SLC) escapes, a decrease in long-latency C-start (LLC) escapes, and reduction in sensorimotor gating as measured by PPI (Fig. 2B). To assess whether dL5-based ablation replicated these deficits, we treated larvae from 2 to 3 dpf with MG-2I, photoablated neurons with 20 min wide-field NIR exposure, and then tested behavior at 6 dpf. dL5-ablated larvae displayed the same deficits as NTR-ablated larvae: increased SLC responses, decreased LLC responses, and decreased PPI (Fig. 2C). This confirms that cytoplasmic dL5 expression coupled with MG-2I treatment and light exposure leads to a lasting loss of zebrafish neurons, resulting in anticipated effects on behavior.

### Ablation of selected dL5-expressing neurons using spatially patterned illumination

**Fig. 3.**
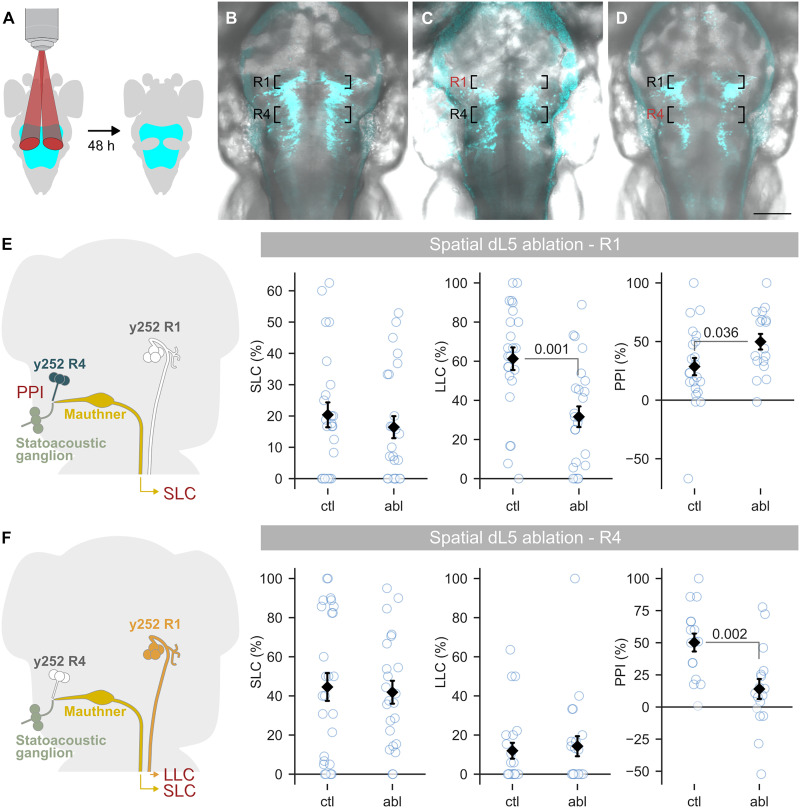
Ablation of dL5-expressing neurons using spatially patterned illumination. (**A**) Patterned illumination from a DMD used to selectively illuminate part of the *y252-Gal4* expression pattern. Larvae were tested for behavior 48 hours after MG-2I treatment and photoablation. (**B** to **D**) Confocal 40-μm substack maximum projections showing dL5-mCer expression and differential interference contrast background for 5-dpf untreated *y252-Gal4, UAS:dL5-mCer* larva (B) and larva 48 hours after selective illumination of R1 (C) or R4 (D) neurons. Scale bar, 100 μm. (**E** and **F**) SLC and LLC escape behavior and PPI in *y252-Gal4, UAS:dL5-mCer* larvae after selective photoablation of R1 neurons (E) or R4 neurons (F). MWU, significant *P* values indicated. The increase in PPI in (E) was not present in replication experiment [analysis of variance (ANOVA), main effect treatment *F*_1,54_ = 2.9, *P* = 0.094 for combined dataset]. In (E), *n* = 24 and 25 for ctl (dL5^−^/Mg2I^+^) and abl (dL5^+^/Mg2I^+^) for SLC and LLC; *n* = 22 and 17 for PPI. In (F), *n* = 27 and 23 for SLC; *n* = 22 and 21 for LLC; *n* = 16 and 17 for PPI.

Distinct neurons in the *y252-Gal4* pattern control escape behavior and PPI. LLC responses require a cluster of *y252* neurons in the rhombomere 1 (R1) pontine tegmentum (*17*), whereas PPI is mediated by glutamatergic neurons in rhombomere 4 (R4) of the medulla oblongata (Fig. 2A) (*16*). The location of neurons that regulate SLC thresholds is unknown. This enabled us to use behavior to assess whether spatially localized dL5 illumination can selectively lesion neuron populations. We used a digital micromirror device (DMD) to project patterned 656-nm NIR light onto defined subsets of *y252*-expressing neurons (Fig. 3A). This approach resulted in selective ablation of either R1 or R4 *y252-Gal4, UAS:dL5-mCer* neurons (Fig. 3, B to D). Consistent with our previous results using laser ablation, selective elimination of R1 *y252*-expressing neurons with light exposure significantly reduced LLC responses but did not diminish SLC or PPI responses (Fig. 3E). Conversely, SLC and LLC responses were not affected by selective R4 *y252* ablation, whereas PPI was substantially reduced (Fig. 3F). This double dissociation confirms that spatially restricted illumination can be used to selectively ablate dL5-expressing neurons of interest. Unlike NTR, which lesions the entire neuronal population defined by a transgenic pattern, spatially selective dL5 ablation addresses this limitation by enabling the precise removal of specific neurons, providing greater control over circuit manipulation for functional studies.

### Presynaptically localized dL5 enables precise ablation of genetically defined synapses

**Fig. 4.**
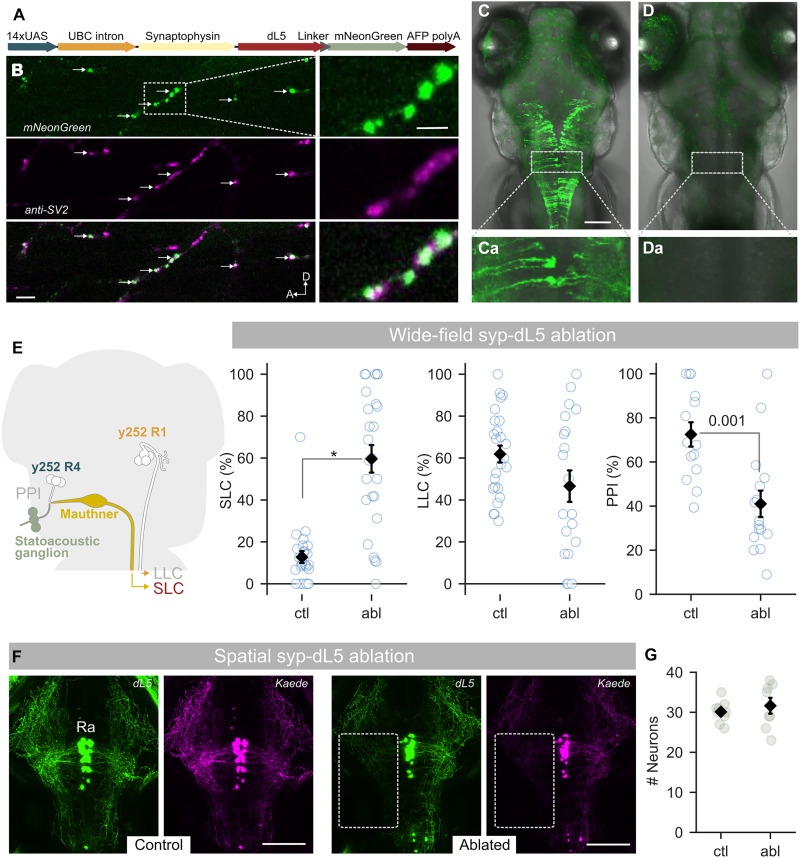
Wide-field ablation using synaptically localized dL5. (**A**) Schematic of the construct used to make the *UAS:syp-dL5-mNeonGreen* transgenic line. (**B**) Horizontal confocal section through the spinal cord of 3-dpf *y417-Gal4, UAS:syp-dL5-mNeonGreen* embryo (green), stained with anti-sv2 (magenta). Scale bar, 20 μm. Arrows show synaptic puncta from *y417* neurons. “A” and “D” denote anterior-dorsal orientation. The boxed region is shown at a higher magnification in the right panel. Scale bar, 5 μm. (**C** and **D**) Maximum projection from the confocal *z*-stack of GFP and differential interference contrast channels in 4 dpf *y252-Gal4, UAS:syp-dL5-mNeonGreen* larvae: untreated control larva (C) and an MG-2I–treated larva exposed for 20 min to wide-field illumination at 3 dpf (D). Scale bar, 100 μm. A square ROI was cropped from each panel in (C) and (D) and is shown at a higher magnification in (Ca) and (Da). (**E**) SLC [*n* = 25 and 25 for ctl (dL5^−^/Mg2I^+^) and abl (dL5^+^/Mg2I^+^)] and LLC (*n* = 25 and 20) escape responsiveness and PPI (*n* = 14 and 16) in control and wide-field light–exposed *y252-Gal4, UAS:syp-dL5-mNeonGreen* larvae. MWU, significant *P* value indicated and *P < 0.001. (**F**) Maximum projection of 80-μm confocal stacks from 4-dpf *tph2:Gal4, UAS:syp-dL5, UAS:Kaede^Red^* untreated larvae and in larvae exposed to NIR illumination in the indicated region of the left hindbrain at 3 dpf. Scale bars, 100 μm. Ra, raphe neurons. (**G**) Number of neurons in dorsal raphe 24 hours after photoablation of neuropil in the region indicated in (F) in untreated [ctl (dL5^−^/Mg2I^+^), *n* = 9] and photoablated larvae [abl (dL5^+^/Mg2I^+^), *n* = 8]. Welch *t* test, *P* = 0.50.

Next, we constructed a synaptically localized version of dL5 through fusion to the synaptic vesicle protein synaptophysin (*20*) and switched the fluorescent tag to mNeonGreen to better resolve small patches of expression (Fig. 4A) (*21*). Like the dL5-mCer cassette, the synaptophysin-dL5-mNeonGreen (syp-dL5) cassette labeled cell bodies and neurites but also showed punctate expression, consistent with synaptic localization (Fig. 4, B and C). Immunostaining with the sv2 antibody, which labels synaptic vesicle protein 2, confirmed that puncta were presynaptic elements (Fig. 4B) (*22*). Treatment of *y252-Gal4, UAS:syp-dL5* larvae with MG-2I and exposure to wide-field illumination led to a near-complete loss of mNeonGreen fluorescence (Fig. 4, C and D). When tested 72 hours after light exposure, larvae reproduced the behavioral phenotypes generated by NTR ablation, including an increase in SLC responses, reduction in PPI, and trend reduction in LLC responses (Fig. 4E). This shows that the syp-dL5 fusion protein retains phototoxicity.

Because neuronal somas were also labeled by syp-dL5, it was possible that the loss of the dL5 signal in the neuropil was secondary to photoablation of cell bodies. Conversely, we could not exclude the possibility that synaptic ablation leads to subsequent degeneration of cell bodies; this would preclude lesioning specific synapses to test their functional relevance. Last, despite the lack of recovery over a 48-hour period, we could not be certain that synapses were eliminated. Presynaptically localized miniSOG was reported to inactivate synapses by locally destroying adjacent vesicle release proteins, resulting in functional recovery after newly synthesized proteins were trafficked back to the synapse (*9*). To assess whether syp-dL5 enabled selective lesioning of synapses, we targeted a neuropil region innervated by raphe neurons in triple transgenic *tph2:Gal4, UAS:syp-dL5, UAS:Kaede* larvae. Because Kaede is cytoplasmic rather than vesicle associated, we reasoned that if synapses were inactivated rather than ablated, most Kaede expression would persist even if dL5-mNeonGreen expression was lost. In addition, because raphe neurons are easily visualized, we could also determine whether synaptic ablation led to cell body degeneration. To distinguish Kaede from mNeonGreen, we first photoconverted Kaede from green to red with wide-field UV light exposure, then mounted larvae for microscopy, and used the DMD to train NIR light on a dense neuropil region in the left hindbrain innervated by raphe neurons (Fig. 4F). When we imaged larvae the next day, syp-dL5-mNeonGreen fluorescence and Kaede^Red^ fluorescence were both almost completely eliminated in the selected region, suggesting that we had not merely photoablated local proteins but destroyed cellular components in the targeted region. Moreover, raphe neurons were intact, demonstrating that the loss of synaptic label can be achieved without damage to cell bodies (Fig. 4G).

### Presynaptic ablation attenuates postsynaptic responses and disrupts behavior

**Fig. 5.**
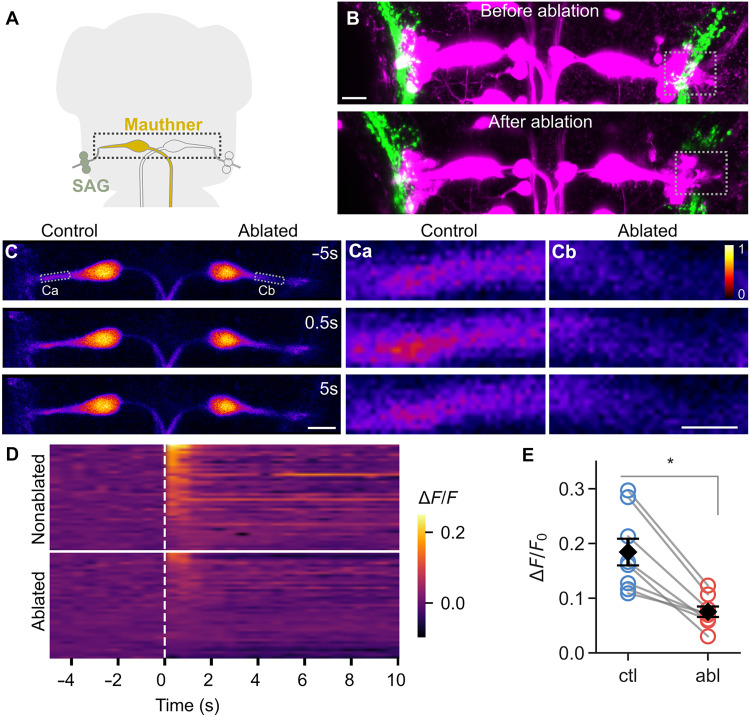
Ablation of SAG synapses impairs evoked postsynaptic activity in the ipsilateral Mauthner lateral dendrite. (**A**) SAG neurons labeled in the *y256-Gal4* line project to the lateral dendrite of the Mauthner cell. The boxed area indicates the region shown in (B). (**B**) Maximum intensity projections of confocal images from a rhodamine-dextran–filled Mauthner cell (magenta) in a *y256-Gal4; UAS:syp-dL5* larva, labeling presynaptic terminals (green). The region of the lateral dendrite selected for targeted ablation in B1 is indicated by a square. The top panel shows before ablation; the bottom panel shows the same fish 24 hours after selective ablation of SAG projections to the right Mauthner cell lateral dendrite (boxed region). Scale bar, 20 μm. (**C**) Single-plane confocal images showing acoustic-evoked calcium responses in the Mauthner neuron filled with Oregon Green-dextran in a *y256-Gal4; UAS:syp-dL5* larva. Images are shown 5 s before, 0.5 s after, and 5 s after acoustic stimulation in a larva where SAG projections to the right lateral dendrite were ablated. [(Ca) and (Cb)] Magnified views of control (left) and illuminated (right) dendrites at the corresponding time points. Scale bars, 20 μm. (**D**) Raster plots showing acoustic-evoked calcium activity in Mauthner cell lateral dendrites for nonablated and ablated sides. Each row represents a single trial, aligned to the onset of the acoustic stimulus (time, 0 s; dashed line). Calcium responses were normalized to the mean prestimulus baseline. (**E**) Quantification of lateral dendrite calcium responses across individual fish. Bars represent mean Δ*F*/*F*_0_ responses to acoustic stimulation for nonablated (blue) and ablated (red) sides, with error bars indicating SEM. Individual points show responses from single fish, and paired lines connect measurements from the same animal. Number of neurons in nonablated [ctl (dL5^−^/Mg2I^+^), *n* = 8] and ablated [abl (dL5^+^/Mg2I^+^), *n* = 8] groups. Wilcoxon signed-rank test, *P < 0.001.

The Mauthner cell receives direct input from statoacoustic ganglion (SAG) neuron afferents (Fig. 5A). To test the functional impact of presynaptic ablation, we used *y256-Gal4; UAS:syp-dL5* transgenic larvae, which labels SAG synapses that decorate the lateral dendrite of Mauthner cells (*15*). We unilaterally ablated SAG presynaptic termini on one Mauthner cell lateral dendrite using patterned NIR illumination and then recorded acoustic-evoked calcium responses bilaterally in dendrites of Mauthner cells that were backfilled with Oregon Green-dextran. Confocal imaging verified the targeted removal of presynaptic terminals in the selected dendritic region that persisted for at least 24 hours, confirming that syp-dL5 expression can be used to ablate specific output synapses from genetically labeled neurons (Fig. 5B). Calcium responses to auditory/vibrational stimuli in Mauthner dendrites on the side where presynaptic terminals had been photoablated were strongly reduced compared to the contralateral, untreated Mauthner dendrites (Fig. 5C). Raster plots of individual trials showed markedly diminished Δ*F*/*F*_0_ transients in the dendrite disconnected from presynaptic input (Fig. 5D), and quantification across multiple larvae demonstrated a significant reduction in peak Δ*F*/*F*_0_ on the ablated side relative to the control side (Fig. 5E). Thus, presynaptic ablation effectively diminishes postsynaptic activity in a directly connected neuron, verifying that the syp-dL5 system can selectively remove defined synaptic connections. This experiment also highlights the potential of combining projection-specific photoablation with calcium imaging to study the causal role of individual presynaptic populations in circuit function.

**Fig. 6.**
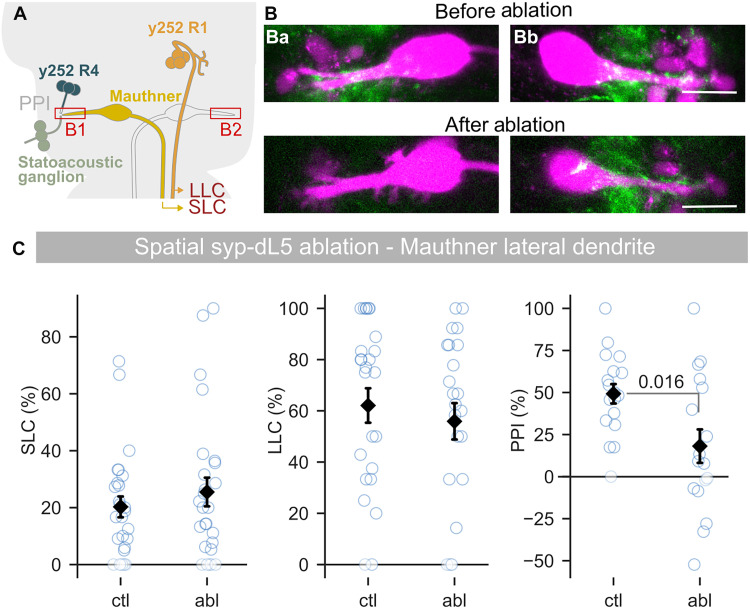
Ablation of *y252* synapses at the Mauthner cell lateral dendrite impairs PPI. (**A**) *y252-Gal4* neurons project to the lateral dendrite of the Mauthner cell. The area of Mauthner cell dendrite illuminated in B1 is indicated. (**B**) Single confocal slice through rhodamine-dextran–filled Mauthner cells (magenta) from an individual *y252-Gal4; UAS:syp-dL5* larva (green) before and 24 hours after illuminating the left (Ba) lateral dendrite. Top panels show both cells before ablation; bottom panels show the same cells after ablation of the left side only. The right side (Bb) was unmanipulated. Scale bars, 20 μm. (**C**) SLC [*n* = 26 and 26 for ctl (dL5^−^/Mg2I^+^) and abl (dL5^+^/Mg2I^+^)] and LLC (*n* = 26 and 23) escape behavior and PPI (*n* = 18 and 17) after bilateral ablation of *y252-Gal4, UAS:syp-dL5* synapses at the Mauthner cell lateral dendrites. MWU, *P* value indicated.

We previously showed that R4 *y252* neurons project to the lateral dendrite of the Mauthner cell (*16*). We found that during PPI, glutamate release from SAG neurons onto the Mauthner cell is depressed, consistent with presynaptic inhibition. Thus, ablating *y252* synapses in this region should disrupt PPI (Fig. 6A). To first test whether we could use patterned illumination to ablate synapses from *y252* neurons, we backfilled the Mauthner cells by injecting Alexa Fluor 658-dextran into the spinal cord in *y252-Gal4, UAS:syp-dL5* larvae. As expected, mNeonGreen puncta decorated the Mauthner cell lateral dendrite (Fig. 6B) (*15*). We then illuminated the lateral dendrite of only the left Mauthner cell. At 24 hours, we imaged the Mauthner cells and saw a loss of mNeonGreen puncta apposed to the left Mauthner lateral dendrite, confirming efficient photoablation of these termini.

To assess whether synaptic photoablation effectively disrupted signaling, we crossed *y252-Gal4, UAS:syp-dL5* to *J1229*, a transgenic line that expresses green fluorescent protein (GFP) in the Mauthner cell, allowing us to visualize the lateral dendrite without injecting dye into the spinal cord (*23*). Subsequently, we illuminated synapses on both left and right Mauthner cell lateral dendrites. In ablated larvae, SLC and LLC responses were unaffected, but PPI was decreased, consistent with a loss of synaptic signaling by *y252* neurons to this area (Fig. 6C). This supports the hypothesis that direct projections from *y252* neurons to this region mediate PPI and demonstrates that synaptically localized dL5 expression can be used to disrupt signaling to specific postsynaptic partners.

## DISCUSSION

A full mechanistic understanding of neuronal circuit function requires decoding critical transmission paths within the central nervous system. This is greatly complicated by the large number of input and output partners associated with any given neuron. Inactivation of selected output synapses from a genetically labeled neuron can help to resolve experimentally how specific neuronal connections contribute to circuit function. To achieve this, we used dL5, a synthetic photosensitizer protein that, when complexed with MG-2I and exposed to NIR, efficiently ablates cells (*12*). We first confirmed that cytoplasmic expression of dL5 in combination with patterned illumination efficiently killed neurons in a region of interest (ROI). Then, by fusing dL5 to the synaptic vesicle protein synaptophysin, we were able to lesion specific output synapses from labeled neurons. To validate this approach, we took advantage of the circuit model for the zebrafish escape response, where we have a relatively advanced understanding of essential neurons, their connections, and associated behaviors. We directly tested the functional impact of projection-specific presynaptic ablation by combining unilateral synaptic ablation with calcium imaging of postsynaptic Mauthner cell dendrites. Selective removal of presynaptic inputs from sensory neurons greatly attenuated sound-evoked calcium responses in the targeted dendrite, providing direct physiological confirmation that syp-dL5–mediated ablation functionally disconnects defined synaptic inputs. Then, we photoablated inhibitory connections from *y252* neurons and found a selective loss of PPI without impairment of other behavioral functions of these neurons, verifying that synaptic ablation can discriminate between output pathways of targeted neurons.

Synaptic ablation serves as a complement to optogenetic systems where light-sensitive proteins in axons or terminals can be photostimulated to trigger or suppress neurotransmitter release, providing a powerful method to assess the functional relevance of specific neuronal connections (*4*). In head-embedded larval zebrafish preparations, precise three-dimensional optogenetic stimulation of individual neuronal processes can be achieved while monitoring behavior, providing powerful temporal control over circuit activity (*24*–*26*). However, such preparations constrain naturalistic movement and rely on transient perturbation of neurotransmission. In addition, synaptically localized optogenetic inhibitors may, in some cases, be difficult to validate or even show counterintuitive consequences because of rebound responses or changes in vesicle release dynamics (*4*, *8*), and direct photostimulation of presynaptically localized channelrhodopsin can drive neurotransmission but may also lead to synaptic depression (*27*). Moreover, while implanted optic fibers can be used to selectively photostimulate terminals in mammals, they are infeasible for small experimental models like zebrafish. Three other systems allow for persistent synaptic inactivation. Illumination of synapses expressing optoSynC or opto-vTRAP components reversibly clusters vesicles, inhibiting release for around 15 or 30 min, respectively (*28*, *29*). These are elegant systems but provide relatively short-term synaptic silencing. This limits their usefulness for studies that require persistent loss of connectivity to measure effects on behavior or circuit function. A method that uses photoactivatable botulinum neurotoxin to cleave the neurotransmitter release protein VAMP2 (vesicle-associated membrane protein 2) provides rapid and persistent interference with signaling but has notable background activity (*30*). These systems are limited by their incomplete suppression of neurotransmission (by around 50%) and their reliance on blue light, which has limited tissue penetration and restricts their utility in behavioral assays that require visual stimulation or at least illuminated conditions. We therefore developed a NIR photosensitizer-based system to selectively ablate synapses from genetically labeled neurons, enabling behavioral experiments in freely moving animals with a light source that does not disturb their visual function. As demonstrated by our synaptic ablation experiments combined with dendritic calcium imaging, in head-restrained larvae, this approach can be directly integrated with single-cell functional readouts to establish causal links between defined presynaptic populations and postsynaptic activity.

We based the syp-dL5 system on earlier work in which presynaptic localization of miniSOG, a small flavin mononucleotide binding protein, efficiently inhibited synaptic transmission (*9*). MiniSOG produces singlet oxygen, leading to the destruction of nearby synaptic release machinery. The dL5 photosensitizer is at least an order of magnitude more efficient for ablation than miniSOG (*13*). In addition, miniSOG is activated by blue light, and flavin mononucleotide is naturally present in cells, raising the risk of ablation under standard experimental conditions. In contrast, the dL5 photosensitizer produces singlet oxygen only during illumination with NIR light in the presence of MG-2I (*12*). This allows experimental subjects to be maintained under normal conditions until a 24-hour treatment period with MG-2I, during which time they remain in dim light. Following NIR exposure, MG-2I is washed out, and subjects can be raised and tested under normal conditions. Synaptic ablation occurs gradually over the next 24 hours after light exposure and persists for several days, offering a substantial time window to experimentally assess the consequences of eliminating neuronal connections.

Persistent projection-specific ablation may be particularly valuable in rodent studies on long-term circuit reorganization involved in plasticity that occurs during learning, recovery from injury, or compensation during chronic disease. However, a limitation of syp-dL5 synaptic ablation in comparison to other optically controlled methods for synapse inactivation is that it is irreversible. Although we expect that, eventually, ablated synapses will be replaced, we have not seen restoration of function over at least a 3-day period. This persistent loss of synaptic connectivity is useful for behavioral studies but limits application for experiments requiring within-subject controls. In addition, unlike other methods where illumination intensity can be used to tune levels of inhibition, synaptic ablation is intrinsically an all-or-nothing phenomenon.

We also demonstrated that transgenic expression of cytoplasmic dL5 can be used to lesion cells in a ROI using spatially patterned illumination, providing a valuable alternative to other methods for targeted cell ablation. Behavioral effects after dL5 ablation were slightly weaker than after NTR ablation despite the relatively complete loss of mCer fluorescence. This may reflect differences in the perdurance of expression of dL5-mCer compared to epNTR-RFP; longer recovery time after photoablation, providing more opportunity for cell replacement during development; or the degree to which expression was variegated in each line. dL5-mediated ablation is similar to a cellular ablation system based on mitochondrially targeted expression of a HaloTag protein in combination with a rhodamine-based photosensitizer dye, which efficiently kills zebrafish neurons over a similar time span (*31*). Neurons are commonly lesioned using a two-photon laser, a technique that provides a very high spatial resolution but that is difficult to calibrate to avoid off-target damage to neighboring cells (*32*). Intersectional genetic strategies can provide exquisitely selective ablation with little or no collateral damage but are limited by the availability of transgenic lines that label cells of interest (*16*, *33*, *34*). On the other hand, photoablation using dL5 should also be carefully validated. Although we found that photoablated neurons did not cause the death of adjacent cells, singlet oxygen generation is likely proportional to transgene expression levels, as well as light intensity and duration, and in some circumstances may be sufficient to cause bystander damage. Similarly, we could not determine whether diffusion of singlet oxygen from syp-dL5–labeled synapses also destroys neighboring termini. However, one experiment suggested that synaptic loss is relatively selective. Auditory afferents from the SAG contact the Mauthner cell lateral dendrite in the same area as *y252* synapses. The finding that SLCs were not affected after ablation of *y252* synapses adjacent to the Mauthner lateral dendrite implies that there was not a substantial loss of auditory termini despite their close proximity.

Our results show that expressing the dL5 photosensitizer at presynaptic sites allows selective and lasting removal of specific synaptic connections, producing both structural elimination of presynaptic terminals and measurable reductions in postsynaptic activity, leading to clear and targeted behavioral changes. By disconnecting defined neural projections for several days, this method complements reversible activity-based approaches and enables the measurement of long-term impacts of synaptic inactivation. This technique should be easily adapted to almost any other circuit with genetically tractable neurons and help to interrogate how unique synaptic connections shape circuit function and behavior in a wide range of systems.

## MATERIALS AND METHODS

### Zebrafish husbandry

All experiments were approved by the National Institute of Child Health and Human Development Animal Care and Use Committee (approval no. 24-007). Experiments were performed on larvae up to 7 dpf before sex differentiation. Larvae were raised at 28°C on a 14:10 light dark cycle in E3 medium supplemented with 1.5 mM Hepes, pH 7.3. Wild-type and transgenic larvae were maintained on a Tüpfel Longfin background acquired from the Zebrafish International Resource Center. Transgenic lines used were *y252Et* (*y252-Gal4*) (*35*), *y417Et* (*y417-*Gal4) (*36*), j1229aGt (*j1229*) (*23*), *Tg(tph2:Gal4)y228* (*tph2:Gal4*) (*37*), *Tg(UAS:Kaede)s1999t* (*UAS:Kaede*) (*38*), and *TgBAC(slc17a6b[vglut2a]:loxP-DsRed-loxP-GFP)nns14* (*vglut2a:DsRed*) (*39*).

### Plasmid construction

To create plasmid UAS:dL5-mCer3 (Addgene plasmid no. 241797), we subcloned the MBIC5-mCer3 cassette from pCS2+ MBIC5-mCer3 (gift from M. Tsang, Addgene plasmid no. 74116; http://n2t.net/addgene:74116; RRID: Addgene_74116) (*12*) into pT1UciMP (Addgene plasmid no. 62215; http://n2t.net/addgene:62215; RRID: Addgene_62215) (*40*). To make a synaptically localized version of dL5, we first replaced *mCer3* in UAS:dL5-mCer3 with zebrafish codon-optimized *mNeonGreen* (*21*, *40*) and then created a fusion with zebrafish *synaptophysin b* (*20*), creating UAS:syp-dL5-mNeonGreen (Addgene plasmid no. 241798). Stable lines were generated by injecting plasmids with codon-optimized tol1 transposase mRNA (*40*).

### Ablations

NTR ablations used *Tg(UAS:epNTR-TagRFPT-utr.zb3)y362Tg* (*UAS:epNTR-RFP*), a variant of NTR engineered for increased ablation efficiency (*18*). Embryos were treated with 10 mM metronidazole (Sigma-Aldrich, M1547) from 3 to 4 dpf under dim light conditions. Controls were siblings from the same clutch that lacked RFP (red fluorescent protein) expression, also treated with metronidazole. For dL5 ablations, we used a stock solution of MG-2I (gift from M. Bruchez, Carnegie Mellon University) or custom-synthesized MG-2I (www.medchem101.com) that was dissolved at 1 mM in ethanol and stored in aliquots at −20°C. Stock was added to E3 medium to make a working solution of 750 nM, supplemented with 300 μM *N*-phenylthiourea (PTU) to inhibit melanophore formation. The working solution was applied to 2-dpf larvae, which were then placed in a dark 28°C incubator. At 3 dpf, the working solution was replaced with E3/PTU medium. For wide-field ablation, six to eight larvae were placed in a single chamber of a 48-well plate containing 1 ml of E3. A 656-nm light-emitting diode (UHP-T-650-EP, Prizmatix) coupled to a 3-mm light guide was used to illuminate the chamber for 20 min (or less for experiments testing the dose-response), providing 550 mW/cm^2^ of power. After treatment, larvae were returned to a regular light-cycle incubator in fresh E3/PTU medium. Controls were non–dL5-expressing larvae that were exposed to MG-2I, handled in parallel to the experimental group including illumination. Larvae were maintained in PTU for behavioral experiments so that we could subsequently verify ablation using confocal imaging.

### Spatial ablation

After anesthesia with tricaine methanesulfonate (0.2 mg/ml; MS-222), larvae were transferred to 100 μl of E3 medium mixed with 200 μl of 2.5% low-melt agarose. Larvae were mounted in a 50-mm glass-bottom dish, covered in E3 medium, and transferred to a microscope with a DMD (Mightex Polygon 400). The same light-emitting diode used for wide-field photoablation was coupled to the Polygon 400 using a 3-mm light guide and controlled via a National Instruments DAQ board. Light was focused on the sample using a 40×/0.8–numerical aperture (NA) water immersion objective, and ROIs were defined using Elements software (Nikon). The power delivered via the DMD was 511 mW/cm^2^, and illumination lasted 20 min. Controls were non–dL5-expressing larvae that were exposed to MG-2I, mounted in agarose in parallel to the experimental group but not illuminated because of time constraints in acquiring sufficient numbers.

### Confocal imaging

Embryos were maintained in E3 medium with 300 μM PTU starting at 24 hours post-fertilization. For imaging at 3 or 4 dpf, larvae were anesthetized in MS-222 for 5 min and subsequently mounted in 2.5% low-melting-point agarose. For low-resolution imaging, larvae were positioned dorsoventrally in 50-mm glass-bottom dishes (no. 1.5), and confocal image stacks were acquired using a laser-scanning confocal microscope (Nikon AXR) equipped with an automated stage and a 20×/1.0-NA objective. For high-resolution imaging, larvae were mounted in three-dimensionally printed plastic inserts, which were placed on Lab-Tak II chambered cover glasses (no. 1.5). Confocal stacks were obtained using either a laser-scanning confocal microscope (Leica TCS SP5 II) with a 40×/0.95-NA water immersion objective or a point scan Nikon microscope with a 40×/1.15-NA objective.

### Histological labeling

To label the Mauthner cell with fluorescent dye, we pressure injected a solution of Alexa Fluor 568–conjugated dextran 10k into the ventral spinal cord of 3-dpf *y252-Gal4, UAS:syp-dL5* larvae (*41*). After 24 hours, we mounted larvae for ablation using the DMD. To photoconvert Kaede from green to red, we exposed larvae to 405-nm ultraviolet light (Formlabs Form Cure) for 5 min. For whole-mount immunohistochemical staining, 3-dpf zebrafish larvae were fixed in 4% paraformaldehyde in phosphate-buffered saline (PBS) at room temperature for 2 hours. Samples were washed three times with PBS, treated on ice with 0.25% trypsin for permeabilization for 5 min, and then blocked with 1% Blocking Reagent (Roche, Indianapolis, US) that was prepared in PBS for 3 hours on a rocker at room temperature. Primary antibody incubation with a mouse anti–SV2 antibody (1:200, Developmental Studies Hybridoma Bank, Iowa City, IA) in PBS-T (PBS and 1% Triton) for 2 days at 4°C. For the secondary antibody, samples were treated with goat anti-mouse Alexa Fluor 633 (1:500, Invitrogen Company, A-21052) in PBS-T overnight at 4°C.

### Microplate measurements

For quantification of Cerulean fluorescence intensity in *y252-Gal4, UAS:dL5-mCer* after photoablation, larvae were placed into a black-walled, round-bottom 96-well plate in medium with PTU and tricaine (one larva per well). For each time point, we recorded 10 readings per well over a 10-min period using a Gen 5 microplate reader (Biotek Instruments). Larvae were then returned to petri dishes and incubated at 28°C until the next time point. Microplate reader filters were set at 430 nm for excitation and 491 nm for emission to detect mCer fluorescence. Data in Fig. 1C shows readings after subtraction of background values recorded from nonfluorescent larvae measured at the same time points.

### Behavioral analysis

To test auditory responses and PPI, we used a National Instruments NI-DAQ card (PCI-6221) to generate 2-ms duration waveforms delivered to larvae using a minishaker (4810, Bruel and Kjaer), as previously described (*42*). PPI is a form of sensorimotor gating in which a weak prestimulus suppresses the SLC response to a subsequent intense stimulus. We used a series of 80 stimuli that comprised a pseudorandom sequence of four types of stimuli separated by 15 s. Stimuli were a weak vibrational stimulus, strong stimulus, weak stimulus with a 50-ms interval before the strong stimulus (PPI-50), and weak stimulus with a 500-ms interval before the strong stimulus (PPI-500). The weak stimulus was 10% of the intensity of the strong stimulus. PPI was calculated as the percentage reduction in SLC responsiveness on PPI trials compared to responsiveness on trials with the strong stimulus alone. For experiments with *y252*, we report (i) SLC responsiveness to the weak stimulus, which increases after ablation; (ii) LLC responsiveness, which decreases after ablation; and (iii) PPI from the PPI-500 (PPI) measurement, which also decreases after ablation (*15*). For SLC responses, we excluded larvae with fewer than 6 of 20 analyzable responses (because of tracking errors, being too close to the wall of the arena, or they were already moving at the time of stimulus presentation). For LLC responses, we used the same criterion, and additional larvae were not included if all their responses were SLC responses. For PPI, we only used larvae with an SLC response frequency of 30 to 95%. Thus, *n*’s for SLC, LLC and PPI are different despite being derived from the same recordings. In some experiments, ablation of *y252* neurons with NTR caused an increase in PPI-50, but this effect was inconsistent between experiments and was not seen with dL5 and the underlying neural mechanism is unknown. Responses were recorded at 1000 frames per second using a high-speed camera, then tracked, and analyzed using Flote software (*42*).

### Calcium imaging

Mauthner neurons were retrogradely filled at 3 dpf in *y256-Gal4, UAS:syp-dL5* larvae with an Oregon Green 488 BAPTA-1 dextran, 10000, calcium indicator to monitor dendritic calcium activity in response to acoustic stimulation. Calcium signals in the lateral dendrites were quantified as the relative change in fluorescence (Δ*F*/*F*_0_), calculated as

$$\Delta F/F_{0}=\left(F-F_{0}\right)/\left(F_{0}\right)$$

where *F* is the fluorescence at each time point, and *F*_0_ is the mean baseline fluorescence during the 0- to 10-s period preceding stimulus onset. Trial-by-trial responses were aligned to the onset of the acoustic stimulus and displayed as raster plots to visualize response variability. For quantification, the peak Δ*F*/*F*_0_ within a 10-s window following stimulus onset was measured for each trial, and responses from the same animal were averaged to yield a single value per dendrite. To assess the effect of unilateral synapse ablation on calcium responses, paired comparisons between dendritic responses on ablated and nonablated sides were performed using the Wilcoxon signed-rank test, a nonparametric test appropriate for small sample sizes and nonnormally distributed data. Means ± SEM are reported for each condition to summarize variability across animals.

### Statistical analysis

Data were analyzed using Python 3.8 and SciPy (*43*). For behavioral experiments comparing startle responsiveness (SLC and LLC) and PPI between control and ablated larvae, values were generally not normally distributed. We therefore compared groups using a two-tailed Mann-Whitney *U* (MWU) test. We used α = 0.05 as the significance threshold. Each larva was tested with multiple stimuli, but results were calculated for each individual before statistical analysis; *n*’s refer to the number of larvae per control or ablated group. In plots of behavioral data, black diamonds and error bars represent the means and standard error, respectively. We provide exact *P* values in graphs except where *P* < 0.001, indicated using an asterisk.
