# Supplementary material for: Functional interrogation of neuronal connections by chemoptogenetic presynaptic ablation
Source: Sci Adv. 2026 Apr 29;12(18):eaeb6755. doi: 10.1126/sciadv.aeb6755 (PMC13127562; doi:10.1126/sciadv.aeb6755)
Supplement: Supplementary file 1 — Figs. S1 to S4 [file sciadv.aeb6755_sm.pdf]

Science Advances
AAAS

Supplementary Materials for

Functional interrogation of neuronal connections by chemoptogenetic presynaptic ablation

Hariom Sharma *et al.*

Corresponding author: Harold A. Burgess, burgessha@mail.nih.gov

Sci. Adv. **12**, eaeb6755 (2026)
DOI: 10.1126/sciadv.aeb6755

This PDF file includes:

Figs. S1 to S4

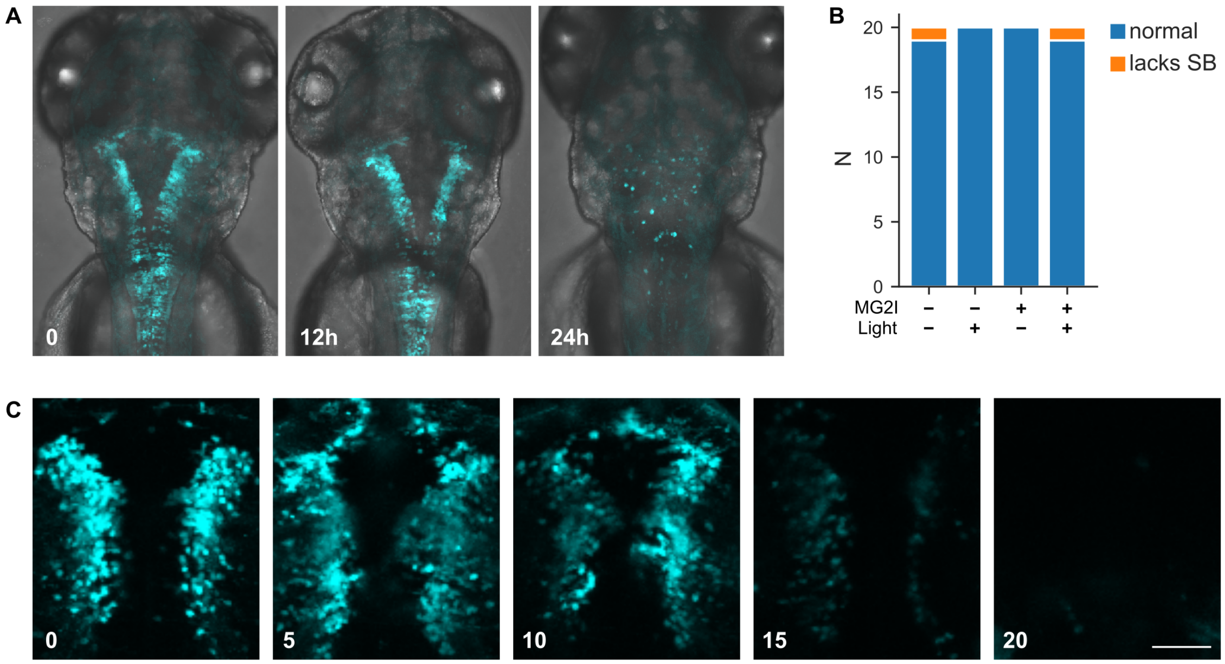

Figure S1. Neuronal ablation using cytoplasmic dL5. A. Maximum projections of confocal images in *y252-Gal4, UAS:dL5-mCer* larva immediately after, and at 12 and 24 hours after exposure to widefield NIR illumination in the presence of MG-2I. **B**. Swimbladder (SB) inflation in groups of non-transgenic larvae treated with MG-2I and/or intense illumination at 3 dpf, then assessed at 6 dpf. N is number larvae. **C**. Maximum projection of 50 µm confocal stacks through the hindbrain of *y252-Gal4, UAS;dL5-mCer* larvae, 24 h after treatment with MG-2I and exposure to widefield NIR illumination for the indicated number of minutes. Scale bar 50 µm.

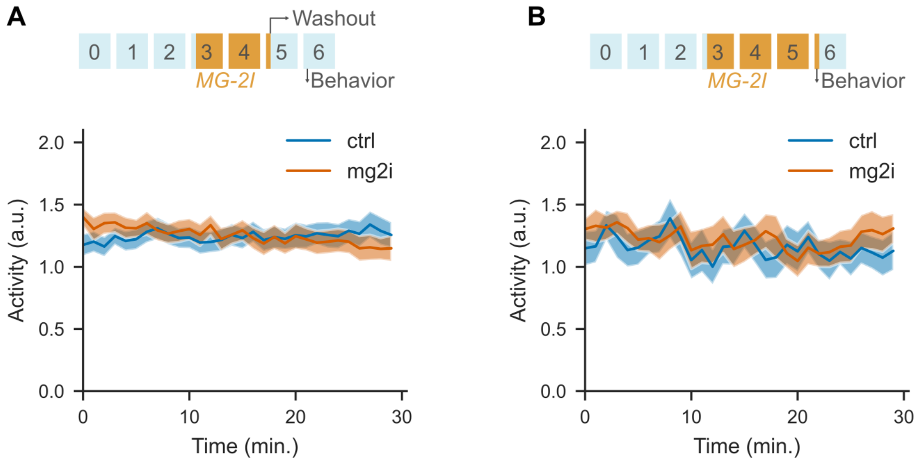

Figure S2: Effect of MG-2I treatment (without NIR exposure) on swimming behavior of transgenic dL5 expressing larvae. A. Larvae were exposed to MG-2I for 2 days. Spontaneous swimming behavior was measured 24h after removal and washout of the drug. No effect of prior drug-exposure was apparent, t-test t[65] = -0.11, p=0.92. **B**. Larvae were treated with MG-2I for 3 days, then swimming behavior assessed while larvae remained immersed in the drug. No effect of drug, t[29] = -0.44, p=0.67.

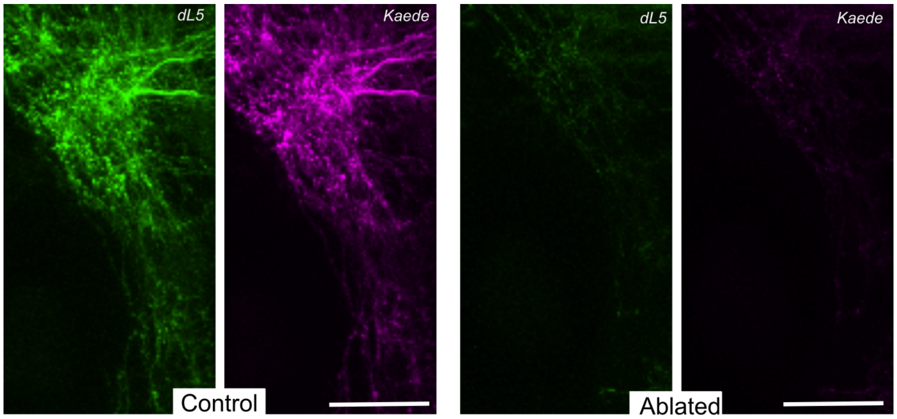

Figure S3. High-magnification view of the left hindbrain region after patterned illumination of syp-dL5 expression larvae. Maximum projection of 80 μm confocal stacks from 4 dpf *tph2:Gal4, UAS:syp-dL5, UAS:Kaede*^{Red} larvae. The indicated region of the left hindbrain shown in Figure 4(F) is shown here at higher magnification to visualize cellular details and the effects of NIR illumination applied at 3 dpf. Scale bar 50 μm.

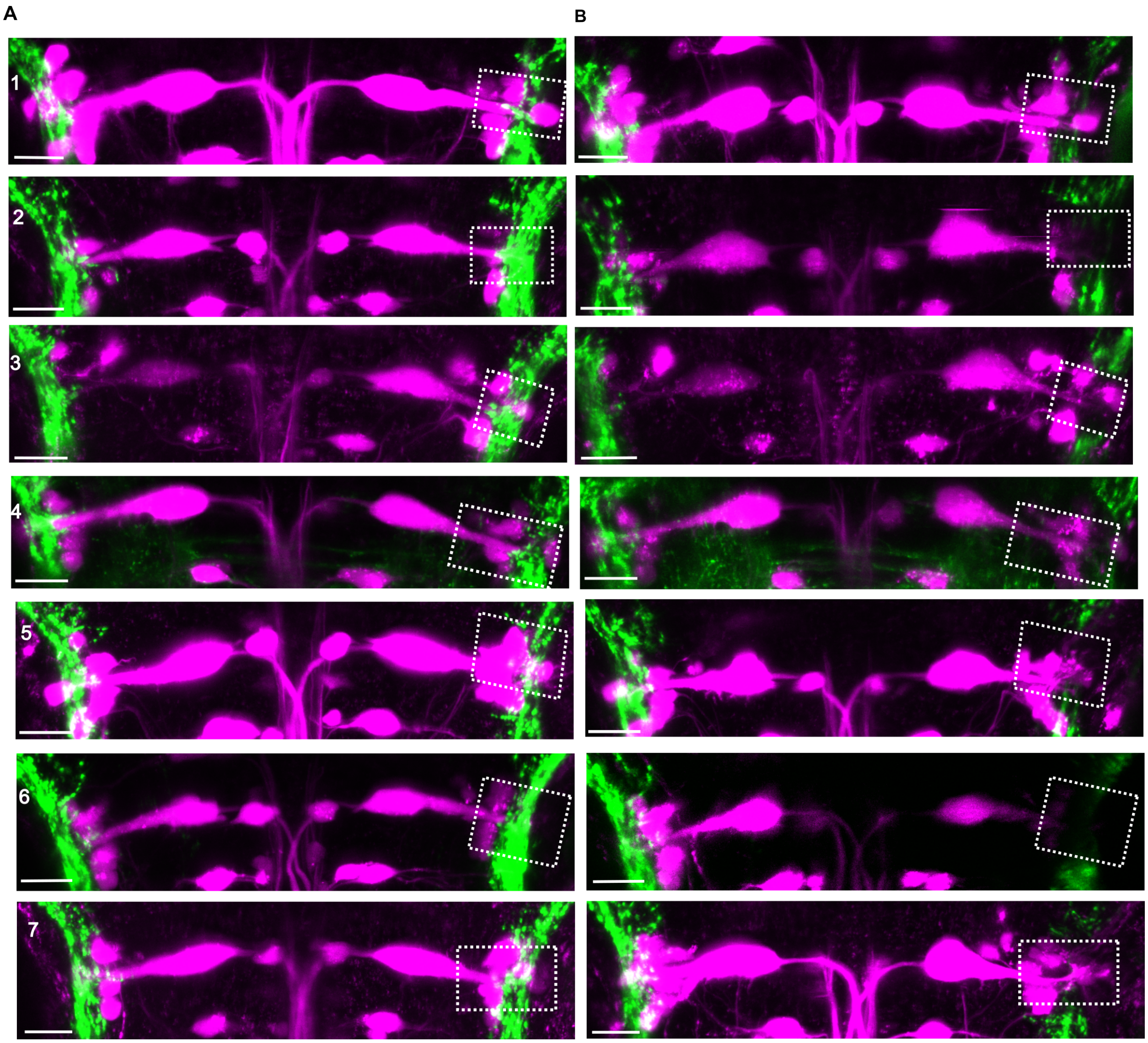

Figure S4. Targeted ablation of SAG neuron projections to the Mauthner cell lateral dendrite region in *y256-Gal4* larvae. A. Representative confocal image of a rhodamine-dextran–filled Mauthner cell prior to targeted ablation. The boxed region indicates the illumination area selected for ablation on the lateral dendrite. **B**. The same larva imaged 24 h after targeted ablation. The boxed region marks the previously illuminated area. Seven individual larvae were imaged before and 24 h after ablation. Scale bar, 20 µm.
